# 100 kHz MAS Proton-Detected NMR Spectroscopy of Hepatitis B Virus Capsids

**DOI:** 10.3389/fmolb.2019.00058

**Published:** 2019-07-24

**Authors:** Lauriane Lecoq, Maarten Schledorn, Shishan Wang, Susanne Smith-Penzel, Alexander A. Malär, Morgane Callon, Michael Nassal, Beat H. Meier, Anja Böckmann

**Affiliations:** ^1^Molecular Microbiology and Structural Biochemistry, Labex Ecofect, UMR 5086 CNRS, Université de Lyon, Lyon, France; ^2^Physical Chemistry, ETH Zurich, Zurich, Switzerland; ^3^Department of Medicine II/Molecular Biology, Medical Center, University Hospital Freiburg, University of Freiburg, Freiburg, Germany

**Keywords:** solid-state NMR, fast MAS, proton detection, carbon detection, deuteration, hepatitis B virus, capsid, core protein

## Abstract

We sequentially assigned the fully-protonated capsids made from core proteins of the Hepatitis B virus using proton detection at 100 kHz magic-angle spinning (MAS) in 0.7 mm rotors and compare sensitivity and assignment completeness to previously obtained assignments using carbon-detection techniques in 3.2 mm rotors and 17.5 kHz MAS. We show that proton detection shows a global gain of a factor ~50 in mass sensitivity, but that signal-to-noise ratios and completeness of the assignment was somewhat higher for carbon-detected experiments for comparable experimental times. We also show that deuteration and H^N^ back protonation improves the proton linewidth at 100 kHz MAS by a factor of 1.5, from an average of 170–110 Hz, and by a factor of 1.3 compared to deuterated capsids at 60 kHz MAS in a 1.3 mm rotor. Yet, several H^N^ protons cannot be back-exchanged due to solvent inaccessibility, which results in a total of 15% of the amides missing in the spectra.

## Introduction

Proton detection at 60 kHz magic-angle spinning (MAS) in 1.3 mm rotors in most cases requires protein deuteration (Andreas et al., 2015). This often sacrifices expression yields in bacteria, and also reduces the number of proteins to be studied to those for which exchangeable protons, most importantly amide protons, can to a large extent be back exchanged from ^2^H to ^1^H. Compared to 60 kHz, MAS frequencies of 100 kHz further average ^1^H dipolar interactions by a factor of ~0.6 (Penzel et al., 2019), improving resolution in fully-protonated systems and allowing to resolve the resonances of small proteins (Cala-De Paepe et al., 2017; Lakomek et al., 2017; Schubeis et al., 2018). Still, comparison of ^1^H linewidths on protonated and deuterated proteins has shown that deuteration improves resolution even at this frequency (Böckmann et al., 2015; Penzel et al., 2015; Xue et al., 2017).

The sequential assignment of protein resonances is a prerequisite in nuclear magnetic resonance (NMR) to further structural studies, and, as in ^13^C-detected spectroscopy, extended sets of pulse sequences have been developed to enable backbone and sidechain assignments of protonated proteins around 100 kHz MAS (Stanek et al., 2016; Higman, 2018). Nevertheless, today, only a handful of fully-protonated proteins were assigned at these spinning frequencies, including the crystalline model protein GB1 (Andreas et al., 2016), a phage capsid assembly (Andreas et al., 2016), the prion domain of HET-s(218-289) (Stanek et al., 2016; Smith et al., 2017), a heptahelical transmembrane protein (Lalli et al., 2017), the HIV-1 capsid (Struppe et al., 2017), the extracellular domain of neonatal Fc receptor (Stöppler et al., 2018), and a carbonic anhydrase of 29 kDa (Vasa et al., 2019). A 26-mer model RNA was also assigned, demonstrating the potential applications for nucleic acids (Marchanka et al., 2018). Except for the phage capsid assembly (Andreas et al., 2016), the ^13^C and ^15^N chemical shifts were available beforehand from solution NMR or ^13^C-detected solid-state NMR for these proteins, as also in the present study.

Here we investigate HBV nucleocapsids formed by multiple copies of the core protein (Cp). Cp forms stable dimers which self-assemble to form the capsid shell. The predominant form consists of 120 Cp dimers arranged in T = 4 icosahedral symmetry. The core protein has a 149-residue assembly domain (Gallina et al., 1989; Birnbaum and Nassal, 1990) named Cp149, and a 34-residue disordered nucleic acid binding C-terminal domain (Nassal, 1992). Residues 140 to 149 form a flexible linker between the N-terminal and C-terminal domains (Watts et al., 2002). For a T = 4 capsid, there are four unique subunit environments, A, B, C, and D, that are occupied by chemically identical but structurally distinct AB and CD dimers. We have recently sequentially assigned the ^13^C and ^15^N resonances of Cp149 capsids, which revealed narrow lines comparable to microcrystalline proteins (Lecoq et al., 2018b) or other viral capsids studied by NMR (Han et al., 2010; Abramov et al., 2015). In this work, we describe the proton-detected sequential assignments of the amide protons of the fully-protonated protein at 100 kHz, using a set of experiments which do in principle not rely on previous knowledge of ^13^C and ^15^N resonances. We compare the completeness of assignments to the ^13^C equivalent spins, discuss the use of protonated pCp149 vs. deuterated dCp149 samples for this protein, and compare spectra recorded on the deuterated protein at 60 and 100 kHz, to conclude on the assets of each rotor size, namely 3.2, 1.3, and 0.7 mm.

## Materials and Methods

### Sample Preparation

Uniformly ^13^C-^15^N labeled fully-protonated pCp149 capsid samples were prepared as previously described in Lecoq et al. (2018b). Preparation of the uniformly ^2^H-^13^C-^15^N labeled dCp149 resulted in a slightly modified expression protocol. Cells from 10 ml inoculated LB medium were collected by centrifugation (4,000 g, 10 min, 20°C) and resuspended in 100 ml of D_2_O-based M9 minimal medium containing 1 g/l of ^15^NH_4_Cl and 2 g/l of deuterated ^13^C-glucose as sole nitrogen and carbon sources, respectively. The deuterated culture was incubated overnight at 37°C, and then transferred into 900 ml of fresh D_2_O-based M9 medium. When the culture reached an OD_600_ of 2.0, protein expression was induced by adding 1 mM IPTG and cells were grown overnight at 25°C. Protonated and deuterated capsids were purified as previously described in Lecoq et al. (2018b), allowing the accessible labile deuterons in the deuterated sample to back-exchange to protons. For fast-MAS NMR measurements, capsids were dialyzed overnight at 4°C in the solid-state-NMR buffer (50 mM TRIS pH 7.5, 5 mM DTT) and about 0.5 mg (Supplementary Table 3) of capsids were filled into 0.7 mm rotors using home-made filling tools (Böckmann et al., 2009) by centrifugation (200,000 g, 14 h, 4°C). A minute amount of saturated 4,4-dimethyl-4-silapentane-1-sulfonic acid (DSS) solution was added to the protein sediment before closing the rotor for chemical shift referencing.

### NMR Spectroscopy and Data Processing

The carbon-detected spectra used for comparison are described in Lecoq et al. (2018b). Shortly, they were acquired on a wide-bore 800 MHz Bruker Avance II spectrometer equipped with a 3.2 mm triple-resonance MAS probe at 17.5 kHz MAS and a sample temperature of 4°C, as determined from the relationship T (°C) = 455–90 ^*^ δ_H2O_ described in Gottlieb et al. (1997), where the water chemical shift (δ_H2O_) corresponds to the supernatant water signal (Böckmann et al., 2009).

The ^1^H-detected spectra at 100–110 kHz were acquired on a wide-bore 850 MHz Bruker Avance III spectrometer equipped with a 0.7 mm triple-resonance MAS probe and referenced to DSS. The magic angle was set using a 0.7 mm rotor with glycine ethyl ester, optimizing intensity, and *J*-coupling-based splitting of the CO resonance (Penzel et al., 2018). The MAS frequency was set to 100 kHz and the VT gas temperature to 273 K using a nitrogen gas flow of 400 l/h, corresponding to a sample temperature of 22°C, extrapolated from the water chemical shift in a ^1^H 1D (Gottlieb et al., 1997; Böckmann et al., 2009) as detailed above. On the uniformly ^13^C-^15^N labeled fully-protonated sample, a set of four three-dimensional (3D) spectra (hCANH, hCONH, hCAcoNH, hncaCBcaNH) (Penzel et al., 2015) and one two-dimensional (2D) fingerprint spectrum (hNH) were recorded. For comparison, 2D hNH and hCANH spectra with virtually identical acquisition parameters (except for the MAS frequency which was set to 110 kHz in the hCANH) were recorded on dCp149 capsids. Acquisition parameters are given in Supplementary Table 1.

The 2D hNH and 3D hCANH (Barbet-Massin et al., 2014; Penzel et al., 2015) ^1^H-detected spectra at 60 kHz on dCp149 were acquired on a narrow-bore 700 MHz Bruker Avance II spectrometer equipped with a 1.3 mm triple-resonance MAS probe and referenced to DSS. The MAS frequency was set to 60 kHz and the VT gas temperature to 248 K using a nitrogen gas flow of 1,400 l/h, corresponding to a sample temperature of 16°C, extrapolated from the water chemical shift in a ^1^H 1D (Gottlieb et al., 1997; Böckmann et al., 2009) as detailed above. Acquisition parameters are given in Supplementary Table 2.

All spectra were processed using TopSpin 4.0.3 (Bruker Biospin) by zero filling to no more than double the number of acquired points. Spectra were apodized in the direct and indirect dimensions with a shifted sine-bell window function (SSB = 3), except for the determination of linewidths, where no apodization was applied. Spectral analyses and resonance assignments were performed using CcpNmr Analysis 2.4.2 (Vranken et al., 2005; Stevens et al., 2011). Peak positions, linewidths, and peak intensities were fitted using the parabolic fit function integrated in CcpNmr. The standard deviations of the average linewidths were calculated by the square root of the difference between the individual linewidths minus their mean value squared, divided by the number of lines minus one.

For comparison of sensitivities of spectra from 3.2 mm (thin-wall), 1.3 mm, and 0.7 mm rotors, we calculated the total mass by weighting the empty and full rotors (Supplementary Table 3).

The ${\text{T}}_{2}^{\prime }$ bulk relaxation times were measured using a Hahn-echo inserted after an hNH dipolar-coupling based polarization transfer sequence. The resulting proton-detected bulk amide signal has been recorded for different variable delay points. The peak area of each signal was extracted using TopSpin and then exported to MATLAB (MATLAB 2016R, The MathWorks Inc., Natick, MA 2016), where the relaxation series was fitted with a mono-exponential decay function. The fit error was derived using a bootstrapping approach.

### Assignment Deposition

^1^H^N^ chemical shifts of the fully-protonated ^13^C-^15^N-labeled Cp149 capsids at 100 kHz MAS were deposited in the Biological Magnetic Resonance Data Bank (BMRB) under accession number 27845. ^15^N, ^13^*C*', ^13^Cα, and ^13^Cβ chemical shifts, for which some resonances slightly differ from ^13^C-detection based assignments (BMRB 27317 Lecoq et al., 2018b), were deposited as well.

## Results and Discussion

### Sequential Assignments of the Amide Proton Resonances

**Figure 1:**
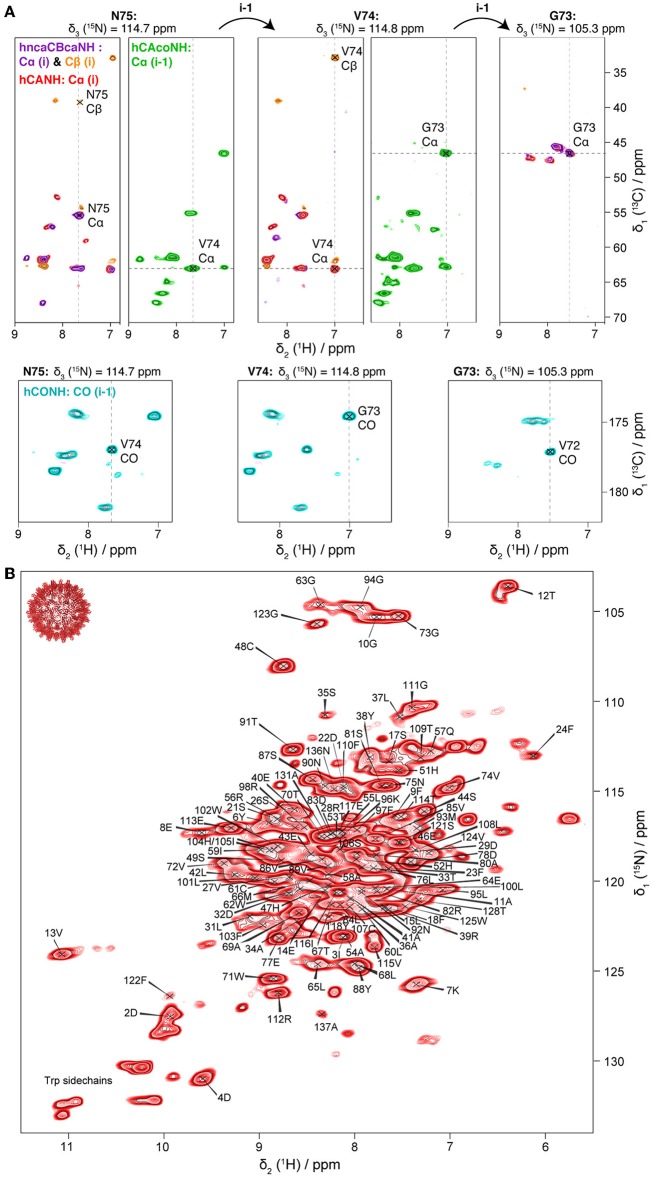
**(A)** 3D planes of pCp149 capsids spectra showing a sequential walk for residues N75, V74, and G73. Four spectra were recorded: hncaCBcaNH in purple (positive peaks: Cα) and orange (negative peaks: Cβ); hCANH in red (Cα); hCAcoNH in green (Cα of the preceding residue); and hCONH in cyan (CO of the preceding residue). **(B)** Assigned hNH spectrum of pCp149 capsids, with resonance frequencies deposited in the BMRB under accession number 27845. The spectrum was processed with QSINE three in both dimensions.

For amide proton assignments, the pCp149 capsid sample was used. Backbone atoms were assigned using four 3D spectra: hCANH showing intra-residue connections; hCONH and hCAcoNH showing inter-residue connections, and hncaCBcaNH connecting the Cβ (and Cα) to the NH. Representative extracts of the spectra are shown in Figure 1A. The four spectra allowed to assign 90% of the H^N^ spins of the protein for residues 1–139 (the 10 residues from the linker are excluded from the statistic analysis as they are not visible in the NMR spectra). Using only the proton-detected spectra shown in Figure 1, 83% of N (including prolines), 91% of Cα, 23% of Cβ, and 80% of C′ could be assigned. Peak assignments are summarized as the mean chemical shift from the combined 3D experiments, and back-predicted on the 2D hNH spectrum in Figure 1B.

### Comparison to ^13^C-Detection-Based Resonance Assignments and Signal-to-Noise

We have recently assigned the ^15^N and ^13^C backbone and sidechain resonances of pCp149 capsids using ^13^C-detected solid-state NMR at 17.5 kHz MAS in a 3.2 mm (thin-wall) rotor (Lecoq et al., 2018b). There, the assignment for residues 1–139 was complete at 97% for N, C′, Cα, and Cβ atoms, and 76% of side-chain heteronuclei. C-terminal residues 140–149 are excluded from the statistics as no cross peaks were detected in either ^13^C- or ^1^H-detected experiments. Except this region, only four residues are missing from the ^13^C-detection-based assignment of HBV capsids. An additional 11 residues compared to the ^13^C-detection based procedure could not be assigned due to low intensity or missing signals in the ^1^H-detected spectra (L16, L19, L30, Q99, V120, S121, R127, P129, Y132, P138, I139, see Supplementary Figure 1). Thus, the ^13^C-^15^N backbone assignment completeness using ^1^H-detection is slightly lower when compared to ^13^C-detection NMR. However, the latter obviously completely lacks information on ^1^H shifts, due to the strong proton line broadening at 17.5 kHz.

**Figure 2:**
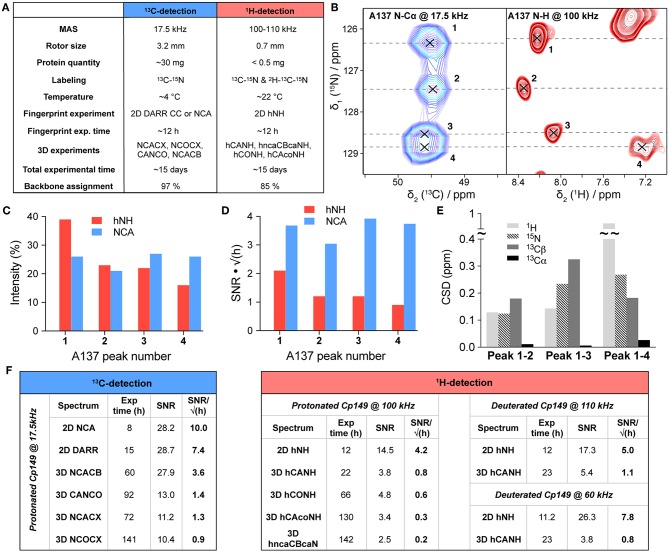
**(A)** Comparison of acquisition parameters and assignments of Cp149 capsids (residues 1–139) using ^13^C-detected experiments recorded in a 3.2 mm rotor (thin-wall) at 17.5 kHz MAS, and ^1^H-detected experiments recorded in a 0.7 mm rotor at 100 kHz MAS. The backbone assignment percentages include ^15^N, ^13^Cα, and ^13^*C*′ resonances. **(B)** Extract of hNH (in red) and NCA (in blue) spectra showing peak splitting for residue A137, which displays four resonances corresponding to the four subunits of the pCp149 capsid. Both 2D spectra were recorded in ~8 h, with 32 scans for the hNH and 8 scans for the NCA. The NCA extract is adapted from previous data (Lecoq et al., 2018b). **(C)** Relative intensities of the four signals of A137 in the hNH and NCA spectra. **(D)** Signal to noise ratio of the four signals of A137 in the hNH and NCA spectra extracted by dividing the peak's height by the noise rmsd and by the square root of the experimental time in hours. A factor of 1.0625 was applied to the NCA spectrum to take into account the field difference (850 MHz for proton-detection vs. 800 MHz for carbon-detection). **(E)** ^1^H, ^15^N, ^13^Cα, and ^13^Cβ CSPs between the four subunit peaks of A137, with $CSD=\left(\frac{\gamma_{X}}{\gamma_{H}}\right)|\delta_{X}|$ (x, nucleus of interest; Δδ, chemical shift difference between the peak of interest and the reference peak, here number one). **(F)** Signal-to-noise ratio (SNR) divided by the square root of the experimental time in hours for ^13^C-detected and ^1^H-detected experiments. SNR was calculated based on the intensities of 14 representative and isolated peaks (D2, D4, G10, T12, V13, D22, E46, C48, W71, D78, T91, C107, G111, and I116) and compared to the global noise of each individual spectrum as estimated in CcpNmr (Vranken et al., 2005). For the DARR, the probe was set to double-resonance mode (^15^N-insert was removed). All carbon-detected spectra were recorded on the same thin-wall 3.2 mm rotor as described in Lecoq et al. (2018b). All proton-detected spectra of pCp149 and dCp149 were recorded on the same rotors. The four rotors were full. A factor of 1.0625 was applied to carbon-detected spectra and a factor of 1.214 to proton-detected spectra at 60 kHz to take into account the field difference (850 MHz for proton-detection at 100–110 kHz vs. 800 MHz for carbon-detection and 700 MHz for proton-detection at 60 kHz). For 3D spectra with multiple correlation peaks, the intensity of the following resonances was taken into account for the SNR calculation: H-N-Cα for the hncaCBcaNH, N-Cα-Cβ for the NCACX (except for Glycines: N-Cα-C′), and N-[C′-Cα]i-1 for the NCOCX. For the deuterated samples, C107 and I116 were excluded from the calculation as they were not back-exchanged.

Experimental parameters used for proton and carbon-detection spectra are compared in Figure 2A. Measurement times are roughly the same between the two approaches for this protein (despite the difference in sample amount of about a factor of one hundred), with about 8–12 h used to record a good 2D fingerprint spectrum (C-C DARR or NCA for carbon-detection, and hNH for proton-detection), and about 2 weeks to record the set of 3D experiments used for the assignment. The reason for the lower assignment completeness in ^1^H-detection lies mainly in the factor of about two lower signal-to-noise ratio (SNR) per square root time of the proton-detected spectra (Figure 2). This brings the divided intensity of some residues with peak doubling or quadrupling (*vide infra*) below the detection limit. In detail, the SNR per square root time calculated on 2D experiments shows a gain by a factor of 1.8 between hNH and DARR-CC spectra, and of 2.4 between hNH and NCA spectra, based on the intensities of 14 representative residues (Figure 2F). The corresponding 3D spectra show an SNR per square root time increase by a factor of 2.3 between the hCONH and the CANCO spectra, and almost a factor of three between the hCAcoNH and the NCOCX spectra for the [Cα]_i−1_ correlation signals (Figure 2F). With respect to the latter, one should note that alternative schemes exist for the hCAcoNH experiment, where J-coupling based transfers (Barbet-Massin et al., 2013) could improve sensitivity under certain conditions. 3D NCACX spectra yield almost twice the SNR per square root time compared to hCANH spectra on the [Cα]_i_ correlation signal, and it additionally provides the carbon sidechain shifts. Globally, the SNR per square root time is therefore still higher within similar experimental time when using 3.2 mm and carbon detection vs. 0.7 mm and proton detection by a factor of about two for both 2D and 3D spectra. Still, especially when available sample amounts prove limiting, the gain in mass sensitivity clearly outweighs the loss in SNR for full rotors for this protein. Indeed, with a total mass here of 0.56 ± 0.04 mg in a 0.7 mm rotor vs. 55 ± 3 mg in a thin-wall 3.2 mm rotor (mass ratio ~100, Supplementary Table 3), and even when considering a loss of roughly a factor of two in SNR, the global gain in mass sensitivity remains ~50 in favor of the ^1^H-detection approach using 0.7 mm rotors. In addition, we compared the SNR for dCp149 capsids at 60 kHz in a 1.3 mm rotor (Figure 2F). We found a similar sensitivity for the 3D hCANH, even if the 2D hNH reveals a better SNR compared to pCp149 capsids at 100 kHz. With a total mass of 4.3 ± 0.1 mg in the 1.3 mm rotor (mass ratio ~8 compared to 0.7 mm), there is therefore no clear advantage of using 1.3 mm rotors rather than 0.7 mm in terms of sensitivity for this protein.

One can ask whether the expected sensitivity effects could be predicted. Data, as well as predictions, related to sensitivity have been described in a recent publication (Mandala and Hong, 2019) where a gain using proton detection of a factor 1.5 was predicted for a 3D spectrum of microcrystalline SH3. As also detailed there, some parameters can be easily compared between the two approaches, as for example the rotor volume (roughly a factor 1/100, *vide supra*), the increased efficiency of the smaller coil which we estimated to scale like the inverse rotor diameters, namely 3.2/0.7 ≈ 4.6 (and 1.3/0.7) ≈ 1.8 (Webb, 1997; Samoson et al., 2010). A factor of eight is gained by detecting protons instead of carbons. A comparison of these parameters results indeed in a factor around 2.5 in favor of carbon detection (for a simple 1D spectrum detected starting with proton equilibrium polarization). In addition, other factors need in principle to be taken into account: for instance, the ratio of the product of the linewidth in all dimensions of the experiment (in Hz), which, as the proton linewidth is often comparable with the carbon linewidth, is on the order of one. Next, the efficiency of the probe circuits, the noise figure of the preamplifier, and efficiencies of the different polarization transfers used during the 2D and 3D experiments, also play a major role. As these values depend highly on the protein, temperature, spectrometer, and/or probe parameters, it is therefore difficult to make good predictions for the relative SNR in proton-detected (0.7 mm) and carbon-detected (3.2 mm) experiments; but for full rotors and to a reasonable approximation they are roughly equal within a factor of two or three.

Finally, ^1^H-detection has the crucial advantage to provide the amide-proton resonances, which are not only sensitive probes for conformational differences, but also for non-covalent interactions such as hydrogen bonds or ring-current effects. We conclude that in cases where enough sample is available, the carbon- and proton-detected approaches are truly complementary. Obviously, when sample amount is limiting, proton detection above 100 kHz is a must, and clearly shows competitive sensitivity.

### Proton Chemical Shifts Are More Sensitive to Detect Capsid Subunits

We have shown that the presence of the A, B, C, D subunits in the icosahedral HBV capsid causes peak splitting in carbon-detected spectra (Lecoq et al., 2018a). This behavior was observed for residues A11-T12, L16-D22, T33-S35, L108-F110, V115, S121-W125, R127-T128, P130-Y132, N136-P140, representing a total of 28 residues over 139 visible ones, i.e., about 20% of the protein. In proton-detected spectra, NMR peak splitting was detected for globally the same residues, including A11-T12, D22, A34-S35, T109, V115, F122-V124, T128, A131, N136-A137, representing a total of 14 residues over 128 possibly visible NH correlations (prolines were removed), i.e., about 11% of the protein. The congruency between the two approaches once more shows that the high sensitivity of NMR to detect asymmetric features in molecular assemblies applies for proton chemical shifts as well. The remaining 9% for which one would also expect NMR peak splitting in the proton spectra are too close to the noise and could therefore not be detected.

For residues whose chemical shifts are different for the four subunits, the proton's high sensitivity led to larger chemical shift differences between the 4 protein subunits. This is illustrated for residue A137 in Figure 2B, which shows extracts of the hNH and NCA spectra of pCp149 capsids, with the signals assigned to the 4 core protein subunits. The respective intensities of the four correlation peaks are displayed in Figure 2C, and show roughly the same distribution in both spectra. The four A137 signals equally show a higher SNR in the NCA than in the hNH when comparing two experiments with a similar experimental time, here about 8 h (Figure 2D). The chemical shift perturbations (CSPs) between the peaks of the four different subunits reveal that, while Cα remains almost unaffected, H^N^, N, and Cβ nuclei are the most sensitive for A137, with ^1^H showing CSPs up to nearly 1 ppm (Figure 2E). This emphasizes the complementarity of the three types of nuclei to measure chemical-shift perturbations as indicator in interaction studies.

### Deuteration vs. Protonation: Incomplete Back-Exchange vs. Increased Proton Linewidths

The choice between deuterated and protonated proteins for NMR studies using proton detection has been lately discussed in the literature (Cala-De Paepe et al., 2017; Linser, 2017; Xue et al., 2017; Schubeis et al., 2018). It has been suggested that MAS frequencies above 100 kHz present an important opening toward the use of fully-protonated proteins, since resolution starts to become high enough to resolve most resonances in 100–200 amino-acid proteins. This allows to bypass the more complex deuterated sample preparation and, even more importantly, back-exchange of amide protons, which can be difficult without a denaturation-renaturation step if they are not solvent accessible. Full protonation also represents an advantage to ease access to side chain resonances. A systematic linewidth comparison for protonated vs. deuterated ubiquitin at 126 kHz MAS is given in Penzel et al. (2019), and predicts significant differences in linewidths (>80 Hz) even for this highest yet described spinning frequency, between protonated and deuterated proteins.

**Figure 3:**
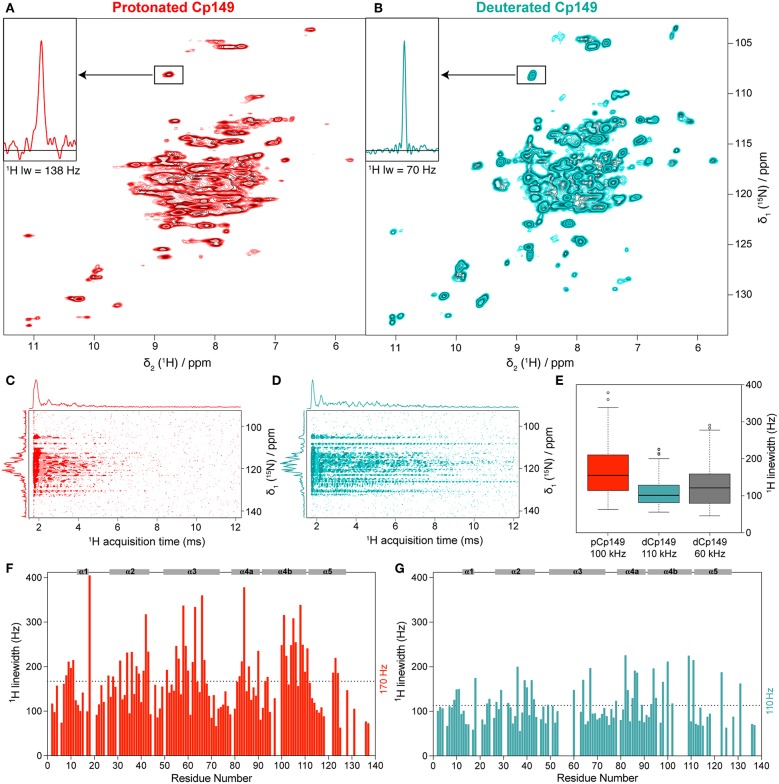
hNH spectra of **(A)** pCp149, and **(B)** dCp149 capsids. The proton line of C48 is shown in the inserts. Both spectra were recorded with identical acquisition parameters (100 kHz MAS, 40 scans, 12 h total experimental time, VTU 273 K, and 25 ms acquisition in the ^15^N dimension) and processing parameters (with no apodization function, cut at 12.9 ms acquisition in the ^1^H dimension and zero-filled to 4,096 points and 1,024 points in the ^1^H and ^15^N dimensions, respectively). **(C,D)** FIDs from hNH spectra processed in the time domain using the xf1 command in TopSpin for Cp149 protonated (red) and deuterated capsids (cyan). **(E)** Dispersion of total proton linewidths measured on all assigned residues in 3D hCANH spectra of pCp149 at 100 kHz (red) and dCp149 capsids at 110 kHz (cyan) and 60 kHz (gray). Median values are indicated as a black line within each box and outliers as circles, defined as exceeding 1.5 times the interquartile range above the third or below the first quartile. **(F,G)** Total proton linewidths for assigned residues observed in the 3D hCANH spectra of pCp149 (red) and dCp149 capsids (cyan) using parabolic fit in CcpNmr (Vranken et al., 2005). Both 3D spectra were run with a similar experimental time of about 22 h and were processed identically (with no apodization function, cut at 12.9 ms acquisition in ^1^H dimension and zero-filled to 2,048 points in ^1^H dimension and 128 points in ^13^C and ^15^N dimensions). The average linewidth value for both samples is indicated as dotted lines and secondary structure elements are identified on the top of each graph.

While here we have used pCp149 capsids for assignments, we wanted to assess which advantages, if any, can be obtained through the use of dCp149. The hNH spectra of the pCp149 and dCp149 capsids are shown in Figures 3A,B. A 1D extract of C48 H^N^ is shown as inset to illustrate the observed difference in proton linewidth on a single resonance. Figures 3C,D show the FIDs of the 2D spectra in the direct acquisition dimension, where the signal decay in the proton dimension can be seen. The signal decay is clearly shorter in the protonated sample than in the deuterated one. In order to quantify, we measured bulk ${\text{T}}_{2}^{\prime }$ relaxation times, which resulted in 2.5 ± 0.1 ms for the protonated sample and 11.6 ± 0.2 ms for the deuterated sample (Supplementary Figure 2). This shows indeed that the homogeneous contribution to the linewidth is smaller by a factor of 4.6 in dCp149 than in pCp149, as is expected by dilution of the strongly coupled proton network with deuterium. One can note that while the proton linewidths are smaller for dCp149, the nitrogen lines appear broader compared to pCp149 by an average factor of 1.2. The same value was found with and without ^13^C decoupling during the acquisition of the indirect dimension.

We also measured a 2D hNH of dCp149 at 60 kHz in a 1.3 mm rotor for comparison, which is shown in Supplementary Figure 3A. The proton linewidths of the three samples were measured for the assigned residues in the 3D hCANH spectra at 60 and 100 kHz for dCp149, and 110 kHz for pCp149. The resulting linewidths are shown in Figures 3E–G and Supplementary Figures 3B,C. The mean proton linewidth was determined to 170 ± 80 Hz for pCp149 at 100 kHz, 110 ± 50 Hz for dCp149 at 110 kHz, and 130 ± 60 Hz for dCp149 at 60 kHz. This results in an average factor of 1.5 between the linewidths of pCp149 and dCp149 at 100–110 kHz, and 1.3 between dCp149 in a 0.7 mm rotor at 100 kHz and dCp149 in a 1.3 mm rotor at 60 kHz. The improvement of 20 Hz between proton linewidths measured on dCp149 at 110 kHz compared to 60 kHz is close to values obtained for deuterated ubiquitin (improvement of 13 Hz in Penzel et al., 2019) and for deuterated GB1 (improvement of only 6 Hz in Cala-De Paepe et al., 2017).

**Figure 4:**
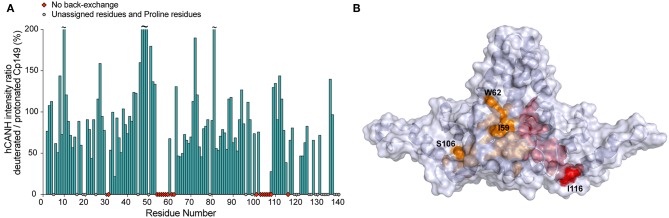
**(A)** Intensity ratio of NMR signals in the 3D hCANH spectra of back-exchanged deuterated Cp149 capsids vs. protonated Cp149 capsids. Unassigned and proline residues are shown as gray circles and residues with no signal in the deuterated sample are shown as red diamonds. The noise was adjusted to the same level for both spectra in CcpNmr before fitting the peak intensities and no normalization was applied. **(B)** Residues whose NH could not be back-exchanged in the deuterated capsids are shown in orange for chain C and in red for chain D on the X-ray structure 1QGT (Wynne et al., 1999).

Despite the better resolution of dCp149 vs. pCp149 at MAS ≥ 100 kHz, it should be mentioned that 15% of the assigned residues are completely missing from the 2D and 3D spectra of the deuterated sample dCp149 due to incomplete back-exchange (Figure 4A and Supplementary Figure 3C). In particular, mainly two regions are affected, namely A54 to T62 and L101 to L108, both located at the base of the spike where the bottoms of helices α3 and α4 interact in the dimer. These residues are buried and thus protected from the solvent, as shown in Figure 4B. Only the sidechains of I59, W62, and I116 are accessible, while the H^N^ remain inaccessible. In HBV capsids, the advantage of deuteration obtained in linewidths must therefore be weighed against the disadvantage of incomplete back-exchange, which obscures information on residues at the center of the capsid spike.

## Conclusions

We have presented here sequential assignments of the amide proton resonances of the fully-protonated HBV capsid using 100 kHz fast MAS solid-state NMR methods. Sequential assignments were obtained from a set of four spectra recorded within 15 days, and yielded 85% of backbone assignments. We compared this assignment to the ^13^C/^15^N assignments obtained previously and found that the ^13^C-detection method led to ~10 % more ^13^C-^15^N assigned residues compared to ^1^H-detection, with the additional advantage that it enabled sidechains assignment using a similar experimental time investment. We have compared the sensitivity of the amide proton resonances to conformational variations as imposed by the icosahedral symmetry, and found that ^1^H chemical shifts are a valuable and sensitive addition to the analysis of ^13^C/^15^N chemical shifts. We showed that, while for the 149-residue HBV core protein a protonated sample allows for assignment of the amide protons, the spectral resolution is still clearly better in a deuterated sample, at both 60 and 100 kHz MAS. This gain in resolution is however concomitant with the loss of a set of amide resonances due to incomplete back-exchange. The success of amide-proton assignments shall open the way for the analysis of the HBV capsid in the presence of partner molecules, notably those only available in small quantities.

## Data Availability

The datasets generated for this study can be found in the Biological Magnetic Resonance Data Bank, 27845.

## Author Contributions

MS, SS-P, LL, AM, and MC conducted the NMR experiments. SW and LL generated protein samples. LL, MS, and AM analyzed the data. MN designed the plasmid and expression/purification protocols. LL, BM, and AB designed and supervised the study, and wrote the manuscript. All authors contributed to the manuscript and approved the submitted version.

### Conflict of Interest Statement

The authors declare that the research was conducted in the absence of any commercial or financial relationships that could be construed as a potential conflict of interest.
