# Supplementary material for: 100 kHz MAS Proton-Detected NMR Spectroscopy of Hepatitis B Virus Capsids
Source: Front Mol Biosci. 2019 Jul 24;6:58. doi: 10.3389/fmolb.2019.00058 (PMC6668038; doi:10.3389/fmolb.2019.00058)
Supplement: Supplementary file 1 [file Presentation_1.pdf]

Supplementary Material

100 kHz MAS proton-detected NMR spectroscopy of hepatitis B virus capsids

Lauriane Lecoq[1#], Maarten Schledorn[2#], Shishan Wang[1], Susanne Smith-Penzel[2], Alexander Malär[2], Morgane Callon[2], Michael Nassal[3], Beat H. Meier[*2], Anja Böckmann[*1]

[#] Contributed equally to the work

[1]Molecular Microbiology and Structural Biochemistry, Labex Ecofect, UMR 5086 CNRS, Université de Lyon, 7 passage du Vercors, 69367 Lyon, France

[2]Physical Chemistry, ETH Zurich, Vladimir-Prelog-Weg 2, 8093 Zurich, Switzerland

[3] University Hospital Freiburg, Dept. of Medicine II / Molecular Biology, Medical Center, University of Freiburg. Hugstetter Straße 55. 79106 Freiburg. Germany

*** Correspondence:**
Corresponding Author
Anja Böckmann a.bockmann@ibcp.fr
Beat H. Meier beme@ethz.ch

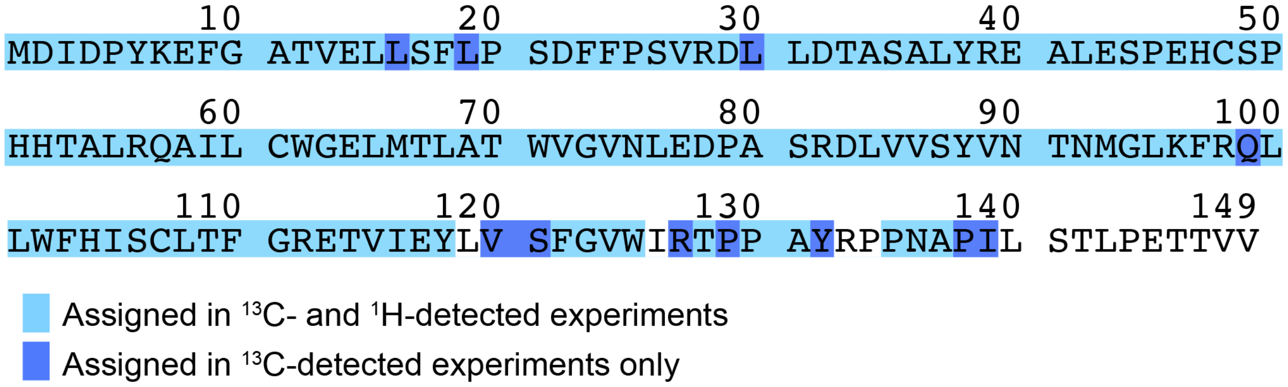

Supplementary Figure 1. Cp149 sequence showing the residues assigned in both ^{13}C- and ^{1}H-detected experiments (light blue) and in ^{13}C-detected experiments only (dark blue). Residues with at least 2 assigned atoms are colored, including prolines in ^{1}H-detected experiments for which carbon resonances could be assigned from the hCONH and hCAcoNH 3D spectra. Unassigned residues are not colored.

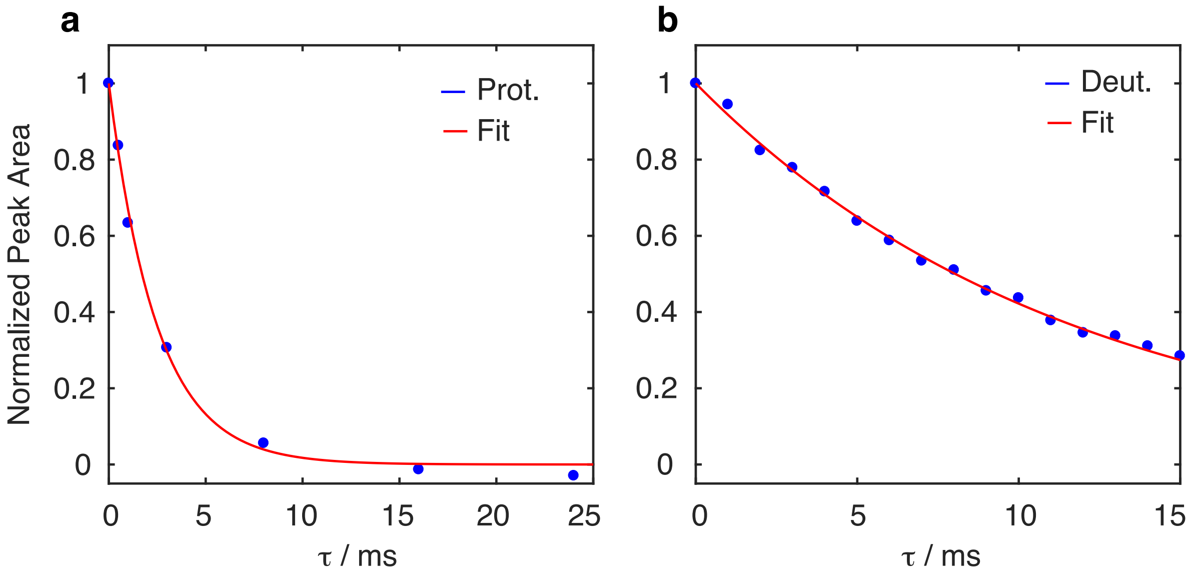

Supplementary Figure 2. T2' decay for the **a)** pCp149 capsids, fitted to a value of $T_2' = 2.5 \pm 0.1$ ms, and for **b)** dCp149 capsids, fitted to a value of 11.6 ± 0.2 ms. Note that these values give the homogeneous, but not the full contribution to the signal decay for the two samples, as depicted in the main text in Figure 3c/d.

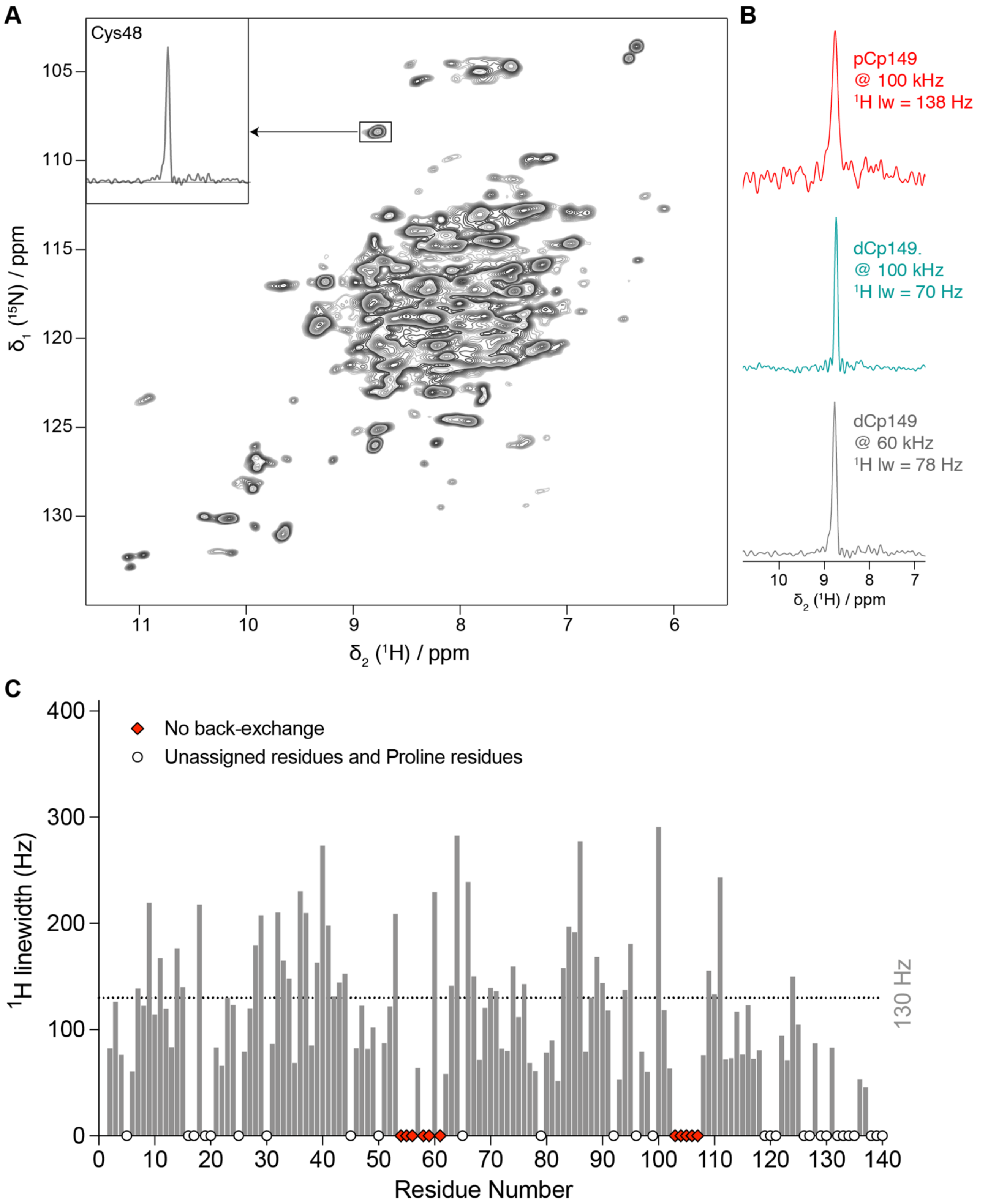

Supplementary Figure 3. (A) hNH spectrum recorded in a 1.3 mm rotor of dCp149 capsids, with the proton linewidth of the isolated C48 residue shown in the insert. The spectrum was recorded at 60 kHz

spinning frequency at a field of 700 MHz with 80 scans, with similar acquisition and processing parameters as data recorded at 100 kHz MAS (11.2 hours total experimental time, 25 ms acquisition in the ^{15}N dimension, processed with no apodization function, cut at 12.9 ms acquisition in ^{1}H dimension and zero-filled to 4096 points and 1024 points in ^{1}H and ^{15}N dimensions, respectively). **(B)** Comparison of C48 proton linewidths for pCp149 at 100 kHz MAS (in red), dCp149 at 110 kHz (in cyan) and at 60 kHz MAS (in grey). **(C)** Total proton linewidths for assigned residues observed in the 3D hCANH spectra of dCp149 capsids at 60 kHz MAS using parabolic fit in CcpNmr (Vranken et al., 2005). The 3D hCANH spectrum was run with a similar experimental time (22 h) as the hCANH spectra recorded at 100-110 kHz, and was processed identically (with no apodization function, cut at 12.9 ms acquisition in ^{1}H dimension and zero-filled to 2048 points in ^{1}H dimension and 128 points in ^{13}C and ^{15}N dimensions). The average linewidth value is indicated as dotted lines. Unassigned and proline residues are indicated as white circles, and residues with no back-exchange (invisible in the 3D spectrum) as red diamonds.

Supplementary Table 1: NMR parameters of 2D and 3D spectra recorded on protonated Cp149 capsids and deuterated Cp149 capsids at 100 kHz and 110 kHz.

	pCp149					dCp149	
**Experiment**	**CP hNH 2D**	**hCANH 3D**	**hCONH 3D**	**hCAcoNH 3D**	**hncaCBcaNH 3D**	**CP hNH 2D**	**hCANH 3D**
MAS frequency / kHz	100	100	100	100	100	100	110
Field / T	20.0	20.0	20.0	20.0	20.0	20.0	20.0
t1 increments	776	64	42	82	112	776	64
Sweep width (t1) / ppm	180	30	16	30	55	180	30
Acquisition time (t1) / ms	25	5	6.1	6.4	4.8	25	5
t2 increments	1024	38	38	48	64	1024	43
Sweep width (t2) / ppm	46.7	35	35	35	35	46.7	40
Acquisition time (t2) / ms	13	6.3	6.3	8.0	10.6	13	6.2
t3 increments	-	1024	1024	1024	1024	-	1024
Sweep width (t3) / ppm	-	46.7	46.7	46.7	46.7	-	46.7
Acquisition time (t3) / ms	-	12.9	12.9	12.9	12.9	-	12.9
^{1}H dec. (swfTPPM) / kHz	10	10	10	10	10	10	10
^{15}N dec. (WALTZ64) / kHz	5	5	5	5	5	5	5
^{13}C dec. (WALTZ64) / kHz	-	5	5	5	5	-	5
180° ref. pulse t1 / channel	^{13}C	^{15}N	^{15}N	^{15}N	^{15}N	-	^{15}N
180° ref. pulse t2 / channel	-	^{13}C	^{13}C	^{13}C	^{13}C	-	^{13}C
Water sup. (120 ms) / kHz	20	20	20	20	20	20	20
Interscan delay / s	1.2	2.0	1.0	1.3	1.3	1.2	1.1
Number of scans	40	16	128	80	48	40	24
Experiment time	**12 h**	**23 h**	**2 days 18 h**	**5 days 10 h**	**5 days 22 h**	**12 h**	**23 h**
**Transfer I**	**HN (dipolar)**	**HC (dipolar)**	**HC (dipolar)**	**HC (dipolar)**	**HN (dipolar)**	**HN (dipolar)**	**HC (dipolar)**
^{1}H field / kHz	78	72	80	77	79	69	76
X field / kHz	17	16	15	15	15	15	16.5
Shape	Tangent ^{1}H	Tangent ^{1}H	Tangent ^{1}H	Tangent ^{1}H	Tangent ^{1}H	Tangent ^{1}H	Tangent ^{1}H
Carrier ^{13}C / ppm (*)	-	56	174	56	-	-	51
Time / ms	1.0	1.2	0.8	1.0	1.0	2.5	4.4
**Transfer II**	**NH (dipolar)**	**CN (dipolar)**	**CN (dipolar)**	**CC (DREAM)**	**NCA (scalar)****	**NH (dipolar)**	**CN (dipolar)**
^{1}H field / kHz	78	-	-	-	-	69	-
^{13}C field / kHz	-	71	66	49	-	-	63
^{15}N field / kHz	17	25	35	-	-	15	36
Shape	Tangent ^{1}H	Tangent ^{13}C	Tangent ^{13}C	Tangent ^{13}C	-	Tangent ^{1}H	Tangent ^{13}C
Carrier ^{13}C / ppm	-	56	174	130	42	-	51
Time / ms	1.0	10.0	20.0	9.0	22	2.5	18.0
**Transfer III**		**NH (dipolar)**	**NH (dipolar)**	**CN (dipolar)**	**CACB (scalar)**		**NH (dipolar)**
^{1}H field / kHz		74	84	-	-		74
^{13}C field / kHz		-	-	60	-		-
^{15}N field / kHz		15	15	36	-		16
Shape		Tangent ^{1}H	Tangent ^{1}H	Tangent ^{13}C	-		Tangent ^{1}H
Carrier ^{13}C / ppm		-	-	174	42		-
Time / ms		1.0	1.6	12.0	7		2.5
**Transfer IV**				**NH (dipolar)**	**CBCA (scalar)**		
^{1}H field / kHz				77	-		
^{15}N field / kHz				15	-		
Shape				Tangent ^{1}H	-		
Carrier ^{13}C / ppm				-	42		
Time / ms				1.2	7		
**Transfer V**					**CN (scalar)**		
Carrier ^{13}C / ppm					42		
Time / ms					22		

Transfer VI	NH (dipolar)
^{1}H field / kHz	79
^{15}N field / kHz	15
Shape	Tangent ^{1}H
Time / ms	1.0

(*) Carriers for ^{1}H and ^{15}N were 4.8 and 117.5 ppm respectively, for all experiments.
dec. refers to decoupling and ref. to refocusing pulses
(**) In deuterated Ubiquitin, the transfer efficiencies have been determined to 0.22 for N-CA-N INEPT out-and-back transfer, and to 0.39 for a single NCA dipolar transfer (Penzel et al., 2015) Thus, if one considers that the N-CA transfer efficiencies are comparable in the protonated sample, the two options are comparable, and actually the INEPT is slightly better. We thus here selected the INEPT out-and-back transfer. Alternatively, the NCA could be implemented as a dipolar CP transfer.

Supplementary Table 2: NMR parameters of 2D and 3D spectra recorded on deuterated and back-exchanged Cp149 capsids at 60 kHz.

	dCp149	
**Experiment**	**CP hNH 2D**	**hCANH 3D**
MAS frequency / kHz	60	60
Field / T	16.4	16.4
t1 increments	248	58
Sweep width (t1) / ppm	70	30
Acquisition time (t1) / ms	25	5.4
t2 increments	1800	40
Sweep width (t2) / ppm	99.2	40
Acquisition time (t2) / ms	13	7
t3 increments	-	1800
Sweep width (t3) / ppm	-	99.2
Acquisition time (t3) / ms	-	13
^{1}H dec. (WALTZ16) / kHz	10	10
^{15}N dec. (WALTZ16) / kHz	5	9
^{13}C dec. (WALTZ16) / kHz	10	10
180° ref. pulse t1 / channel	^{13}C	^{15}N
10 kHz WALTZ16 t2 / channel	-	^{13}C
Water sup. (100 ms) / kHz	15	15
Interscan delay / s	2	1
Number of scans	80	32
Experiment time	**11.2 h**	**23 h**
**Transfer I**	**HN (dipolar)**	**HC (dipolar)**
^{1}H field / kHz	100	85
X field / kHz	40	50
Shape	Ramp ^{1}H	Ramp ^{1}H
Carrier ^{13}C / ppm (*)	-	54
Time / ms	0.6	5
**Transfer II**	**NH (dipolar)**	**CN (dipolar)**
^{1}H field / kHz	100	-
^{13}C field / kHz	-	34
^{15}N field / kHz	43.8	26
Shape	Ramp ^{1}H	Tangent ^{15}N
Carrier ^{13}C / ppm	-	54
Time / ms	0.6	7
**Transfer III**		**NH (dipolar)**
^{1}H field / kHz		95
^{13}C field / kHz		-
^{15}N field / kHz		44
Shape		Ramp ^{1}H
Carrier ^{13}C / ppm		-
Time / ms		0.6

(*) Carriers for ^{1}H and ^{15}N were 4.8 and 120 ppm respectively.
dec. refers to decoupling and ref. to refocusing pulses

Supplementary Table 3: Rotors volumes and weights. The active volume of each rotor was calculated using the inner rotor area based on the inner diameter as indicated by Bruker, multiplied by the coil length. For the 3.2 mm rotor weight, an average was calculated on 3 empty rotors and 4 full rotors filled with similar core protein samples which give a standard deviation of 3 mg. For the weight of 0.7 mm and 1.3 mm rotors, the original rotor samples were used. The mass ratios with respect to the protonated 0.7 mm rotors are given in the right column. All samples were filled using overnight ultracentrifugation at 200 000 g, and residual protein was observed in the funnel for all samples, indicating that the rotors were full. Note that the actual protein quantity required to fill each rotor is about half of the total sample weight indicated, as the sedimented capsids contains solvent. Typical starting amounts of capsids used to fill a rotor are in our hands ~30 mg for a thin-wall 3.2 mm rotor, ~2 mg for a 1.3 mm rotor and ~0.4 mg for a 0.7 mm rotor.

**Cp149**	Active volume (µl)	Empty weight / mg	Full weight / mg	**Total sample weight / mg**	**Mass ratio to 0.7 mm protonated**
**0.7 mm, protonated**	0.37	6.10 ± 0.04	6.66 ± 0.01	**0.56 ± 0.04**	**1**
**0.7 mm, deuterated**	0.37	6.12 ± 0.01	6.67 ± 0.01	**0.55 ± 0.01**	**1**
**1.3 mm, deuterated**	1.45	35.6 ± 0.1	39.9 ± 0.1	**4.3 ± 0.1**	**7.8**
**3.2 mm thin-wall, protonated**	33	394 ± 2	449 ± 3	**55 ± 3**	**98.2**

Supplementary References

Lecoq, L., Wang, S., Wiegand, T., Bressanelli, S., Nassal, M., Meier, B. H., et al. (2018). Solid-state [13C-15N] NMR resonance assignment of hepatitis B virus core protein. *Biomol. NMR Assign.* 12, 205–214. doi:10.1007/s12104-018-9810-y.

Penzel S, Smith AA, Agarwal V, Hunkeler A, Org M-L, Samoson A, Böckmann A, Ernst M, Meier BH. Protein resonance assignment at MAS frequencies approaching 100 kHz: a quantitative comparison of J-coupling and dipolar-coupling-based transfer methods. J. Biomol. NMR. Springer Netherlands; 2015 Oct;63(2):165–86

Vranken, W. F., Boucher, W., Stevens, T. J., Fogh, R. H., Pajon, A., Llinas, M., et al. (2005). The CCPN data model for NMR spectroscopy: development of a software pipeline. *Proteins* 59, 687–696. doi:10.1002/prot.20449.
